# Aged B lymphocytes retain their ability to express surface markers but are dysfunctional in their proliferative capability during early activation events

**DOI:** 10.1186/1742-4933-5-15

**Published:** 2008-11-17

**Authors:** Anthony Blaeser, Kiley McGlauchlen, Laura A Vogel

**Affiliations:** 1 Department of Biological Sciences, Illinois State University, Normal, IL, USA

## Abstract

**Background:**

Ageing is associated with dysfunction in the humoral response leading to decreased protection against infectious diseases. Defects in T cell function due to age have been well characterized but it is unclear if dysfunctions in antibody responses are due to deficiencies in a helper environment or intrinsic B cell defects. Previous studies from our laboratory have shown that aged B lymphocytes are able to differentiate into high affinity antibody-secreting cells at a frequency similar to their young counterparts. However, expansion of B cells *in vivo *was reduced in aged animals when compared to young.

**Methods:**

To further investigate the cause of this reduced expansion, we have now examined early activation events of aged B cells in response to anti-CD40 monoclonal antibody (mAb) and lipopolysaccharide (LPS) stimulation *in vitro*. To do this spleen cells were harvested from young, middle-aged and aged quasi-monoclonal (QM) mice and cultured in complete RPMI for 24 and 48 hours. Cultures contained either LPS or anti-CD40 mAb and murine IL-4. Cells were collected and analyzed using flow cytometry. To examine the proliferative capacity of aged B cells spleen cells were collected as before and cultured in 96 well microtiter plates with either LPS or anti-CD40 mAb and murine IL-4 for 24 hours. Tritiated thymidine ([3H]-Tdr) was added to each well and incubated for another 24 hours after which cells were collected and analyzed using a scintillation counter.

**Results:**

Resting aged B cells exhibited similar levels of CD40 expression when compared to young cells and efficiently up-regulated CD86 and CD69 and also down-regulated CD38 upon stimulation. However, aged B cells proliferated less than young B cells and showed a consistent, but not statistically significant, reduction in their ability to form blast cells.

**Conclusion:**

Aged B cells exhibited a reduced response in some early activation events but produced at least a partial response in all cases. Thus, therapeutic intervention may be possible, despite intrinsically different responses in aged B cells.

## Background

Dysregulation of the humoral immune response has long been associated with ageing, causing inefficient control of many disease-causing microorganisms and cancers, and leading to an increase in morbidity and mortality. These changes are particularly seen in T cell-dependent (TD) immune responses in which aged individuals have shown poorer quality antibodies and lower antibody titers following immunization (reviewed in [1-4]). Similarly, in aged rats following intraduodenal immunization, the number of antigen-specific IgA-secreting cells in the intestinal lamina propria is markedly diminished compared to young animals [5]. However, the numbers of circulating B cells or serum Ig levels are mostly maintained during ageing [6-9]. In fact, the overall number of mature B cells does not seem to be affected by age despite a decline in the number of pre-B cells produced in the bone marrow [10,11]. Bromodeoxyuridine (BrdU) labeling of splenic B cells also suggests an increase in the half-life of mature B cells in aged mice [9]. Likewise, while antibody levels for antigen-specific responses decrease with age, serum immunoglobulin levels, particularly IgG and IgA are reported to be increased [12]. The complexity of a TD immune response and the many cells contributing to this response (e.g. B cells, T cells, macrophages, follicular dendritic cells, etc.) has made it unclear how much of the age-related dysfunction is due to poor helper environment versus intrinsic B cell defects (reviewed in [4,13,14]).

The TD response is initiated in the germinal centers (GC) where isotype-switching, memory cell generation, and clonal expansion occur. This response is impaired in aged animals leading to reduced B cell memory, antibody affinity maturation, and the establishment of long-lived antibody-forming cells (AFCs) [15]. This dysfunction is also reflected in a significant reduction in the number and size of GC following immunization in aged animals [15-17]. Defects have also been reported in the antigen transport mechanisms of follicular dendritic cells (FDC) [16,18,19]. A major function of FDC is to trap and retain antigens, and the development of the FDC-reticulum facilitates the germinal center reaction. Aged mice show a decrease in FDC size and a reduction in numbers of FDC-reticula. Evidence has been found for the requirement of FDC for germinal center formation and thus a decrease in FDC number and size has been associated with a reduced number of germinal centers [16,20].

Successful GC reactions require the interaction of the cell surface protein CD40 with its ligand CD154. CD40 is expressed on virtually all mature B cells as well as a variety of other APCs. This protein plays a key role in the initiation of adaptive immune responses through its interaction with CD154 expressed on activated CD4^+ ^helper T cells (reviewed in [21,22]). Moreover, CD40 mediates multiple biological activities including B cell proliferation, affinity maturation, GC development, rescue of GC B cells from apoptosis, and memory B cell development [23,24]. Whether or not levels of CD40/CD154 expression decrease with age is under debate. It has been reported that there are no age-related decreases in the expression of CD40 on murine splenic cells [8]. However, other reports show that aged human peripheral blood cells have decreased levels of CD40/CD154, while human tonsillar lymphocytes show no significant change in the expression of CD40 due to age [25-27]. Although age-related changes in CD40/CD154 expression or function could contribute to a poor primary and secondary TD response, it is unclear, based on the *in situ *differences in these studies, whether or not age negatively impacts the immune response through decreased expression or function of CD40/CD154.

In adoptive transfer studies, B cells from aged animals were found to be competent to mount a high affinity, isotype-switched response to TD antigens when an appropriate helper environment was provided *in vivo *[4,28]. Using severe combined immunodeficient (SCID) mice as recipients, Yang et al. [28] have shown that antibodies produced by young B cells, when paired with aged T cells, have an altered rate of somatic mutation and V_H _gene usage. When aged B cells were transferred with young T cells into the SCID environment, however, somatic mutations still failed to accumulate normally suggesting intrinsic B cell defects due to age [28]. Previous results from our lab [29] likewise found that upon transfer to young, intact recipients, aged B cells were able to produce a germinal center response, but were unable to expand to the extent of their young counterparts during the primary GC response. It is unclear, however, whether the reduction in expansion was the result of decreased proliferation of the cells within the GC or decreased trafficking to the spleen from blood or other organs [29]. An age-related decrease in migration has been previously noted for lymphocyte migration from the mesenteric lymph nodes to the intestinal lamina propria [30]. A closer look at the decrease in lymphocyte homing has revealed a decrease in the expression of α4β7 and MAdCAM-1, critical markers for homing [31].

To further investigate early activation of aged cells in response to antigenic stimulation, *in vitro *studies were performed. While levels of CD40 were similar on resting aged and young B cells, aged B cells showed a reduced ability to proliferate in response to both anti-CD40 and LPS stimulation. Aged B cells were able to form blast cells upon stimulation, but showed a consistent, but not statistically significant reduction in blast formation. Surprisingly, the induced expression of the early activation markers CD86, CD69 and CD38 up- and down-regulation were found to be normal in aged B cells. In all cases aged B cells produced at least a partial response to stimulation indicating the possibility of improving immunity in the elderly through therapeutic intervention.

## Results

### CD40 expression is equivalent on young and aged freshly isolated B cells

**Figure 1:**
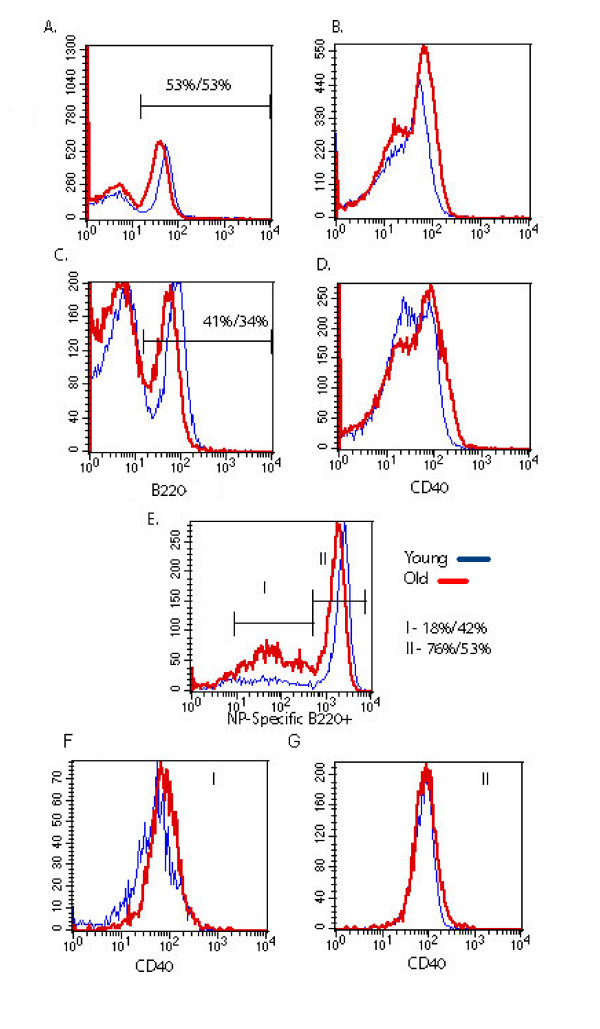
**CD40 levels are similar on freshly isolated B cells from normal and Tg animals**. Freshly isolated SPC from young and aged C57 (A and B) and QM (C-G) mice, were stained with anti-CD40 mAb along with anti-B220 and NP-PE. Percentages of B220^+ ^lymphocytes are shown in (A) and (C), and CD40 expression (B and D) was determined for the B220^+ ^population. Percentages of NP-specific QM B cells and non-NP binding cells are shown in E. The levels of CD40 on NP^- ^and NP^+^/B220^+ ^cells are shown in (F) and (G). Thin lines indicate cells from young animals and thick lines indicate cells from aged animals. Representative of 3 separate experiments.

Age-associated changes in the function or expression of CD40 and CD154 may provide insight to signaling dysfunction within aged B cells. However, there are several contradictory reports concerning the effects of ageing on CD40 expression in B cells [8,25,27], therefore, freshly isolated B cells from young and aged quasi-monoclonal (QM) and C57BL/6 mice were compared for CD40 expression (Figure 1). Both young and aged mice of both strains showed similar levels of CD40 on B220^+ ^B cells, indicating no age-related difference (A and B). Also, for the QM mice, B220^+ ^cells were further analyzed for CD40 expression based on nitrophenyl (NP) – specificity (C and D). As QM mice age, environmental pressures cause the NP-specific B cell populations to undergo V(D)J gene rearrangement and lose specificity for NP [32]. As these cells may have been "antigen-experienced", they may exhibit different levels of CD40. As seen in Figure 1C, QM mice show 42% of the total B220^+ ^splenocyte population is NP^- ^while the young QM show only 18% of the B220^+ ^population to be NP^- ^as expected [33,29]. Upon gating the B220+/NP+ or NP- cells, there was no difference in CD40 expression between aged and young B cells (Fig. 1D). Additional phenotyping (CD69, CD86, CD38, PNA, GL-7, MHCII and CD23: data not shown) of the aged cells was also performed to ensure starting B cell populations were similar. Therefore, both aged and young B cells from normal and transgenic mice express equivalent levels of CD40 and exhibit a resting phenotype.

### Aged B cells express activation markers upon stimulation at a similar level to young B cells

**Figure 2:**
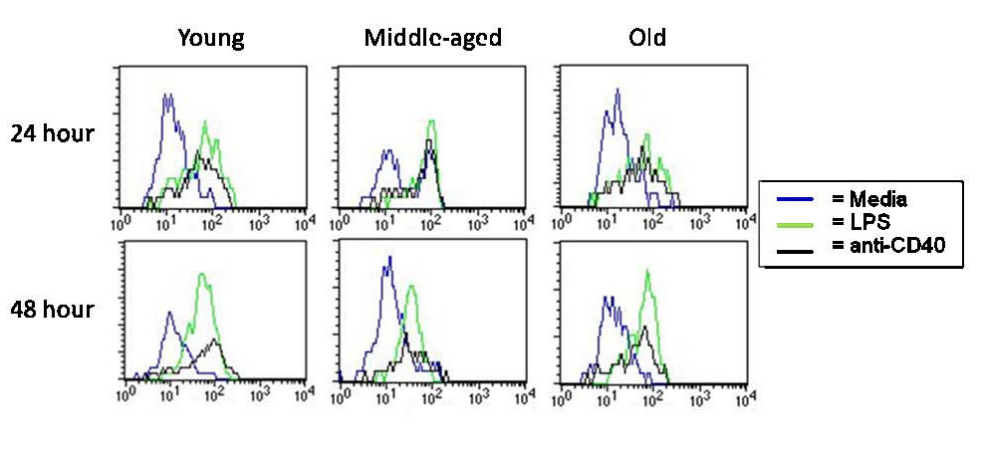
**Splenocytes from aged mice are able to upregulate CD86 effectively upon stimulation**. SPC from young, middle-aged, and aged QM mice were harvested and cultured for 24 and 48 hours. Splenocytes were stained for expression of cell surface markers. Lymphocytes were gated on FSC/SSC and B cells on B220^+^. Representative of 4 separate experiments.

**Figure 3:**
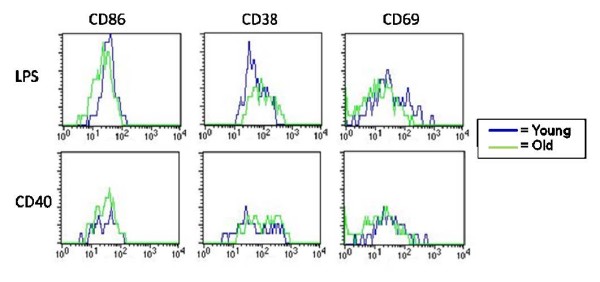
**B cells from aged mice regulate surface marker expression similar to those from young mice**. SPC from young and aged QM mice were harvested and cultured with LPS or anti-CD40 with IL-4 for 48 hours. Splenocytes were stained for expression of cell surface markers. Lymphocytes were gated on FSC/SSC and B cells on B220^+^. Data are representative of 4 separate experiments.

Although levels of CD40 are not affected by ageing, the signaling pathway in aged B cells could be dysfunctional leading to a defect in regulation of early activation markers including CD86, CD69 and CD38. Young and aged freshly isolated QM splenocytes were cultured with media only, LPS, or anti-CD40 mAb with murine IL-4 (interleukin-4) to stimulate B cells in a T cell-independent (TI) and TD manner, respectively. *In vitro *cultures were incubated for 24 and 48 hours then harvested and stained with anti-B220 mAb and one of the following: anti-CD38 mAb, anti-CD86 mAb and anti-CD69 mAb. As shown in Figure 2, splenocytes of all ages were able to upregulate CD86 expression with either stimulus compared to media. Further examination found that both aged and young lymphocytes are also able to effectively upregulate CD69 and down-regulate CD38 upon stimulation with LPS and anti-CD40 mAb (Figure 3). Thus, early regulation of these surface markers appears to be intact in aged B cells.

### Aged B cells form blasts upon stimulation with LPS and anti-CD40 mAb

**Figure 4:**
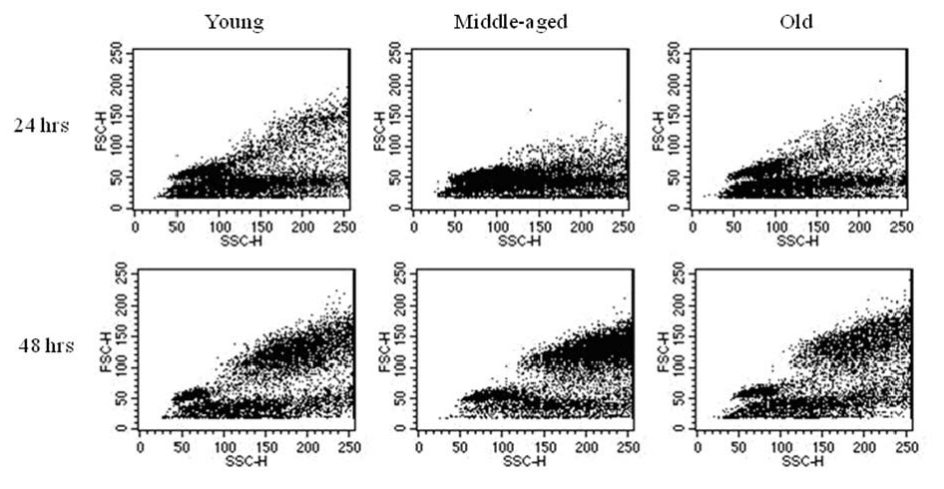
**Aged B cells are able to produce blast cells when stimulated with LPS**. SPC were harvested from young, middle-aged, and aged QM mice and cultured for 24 and 48 hours in stimulating conditions (LPS) and analyzed for side and forward scatter by flow cytometry. Data are representative of 4 experiments.

**Figure 5:**
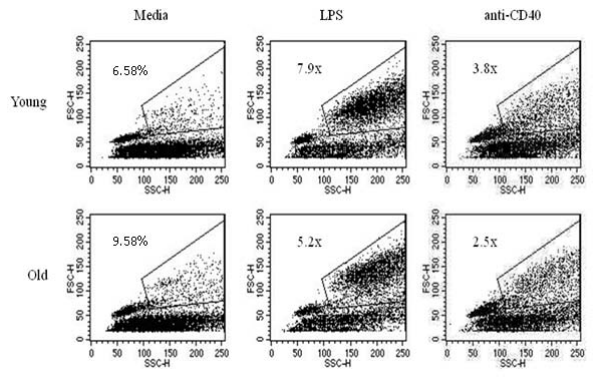
**Aged B cells are able to effectively produce blast cells in response to stimulation**. SPC from young and aged QM mice were harvested and cultured for 48 hours. Blast cells were gated using FSC/SSC. Percentage of blast cells in media were divided into percentages of blast cells for each stimulating condition to give fold-increase as shown.

Another early sign of B cell activation is the formation of blast cells. Splenocytes from young and aged QM mice were cultured for 24 and 48 hrs with media only, LPS, or anti-CD40 mAb as above. Analysis of cells was done using flow cytometry to identify blasts based on forward and side scatter. An increase in blast formation was seen between 24 and 48 hrs for all ages when stimulating conditions were added (Figure 4), thus aged B cells were not intrinsically unable to form blasts. Notably, some individual aged mice (about 1:4 individuals) showed more blast formation when grown in media alone than did young cells (Table 1), so for this reason, background percentages from media only were divided into each of the stimulating conditions to yield "fold increase" of blasts induced through stimulating conditions (Figure 5 and Table 1). Even accounting for increased blast formation in media alone, aged B cells were able to produce blasts under stimulating conditions. Interestingly, a consistent, but not significant, trend of lower antigen-induced responses in the aged cells was observed.

**Table 1:** Aged B cells are able to form blast cells after 48 hours.

		Media		LPS		Anti-CD40	
		% Blast Formation	Fold-increase	% Blast Formation	Fold-increase	% Blast Formation	Fold-increase
Experiment 1	Young	7	__	52	8	25	4
	Aged	10	__	50	5	24	3

Experiment 2	Young	10	__	71	7	32	3
	Aged	20	__	68	3	38	2

SPC from young and aged mice were harvested and cultured for 48 hours in media only, LPS, or anti-CD40 mab with murine IL-4. Blast cells were gated as in figure 5 and percent blast cells were calculated. Percent of blasts from stimulating conditions were divided by percent of blast cells in media only to produce a fold-increase. Data is representative of 3 separate experiments.

### Aged B cells show a decreased proliferation upon stimulation with LPS and anti-CD40 mAb

**Figure 6:**
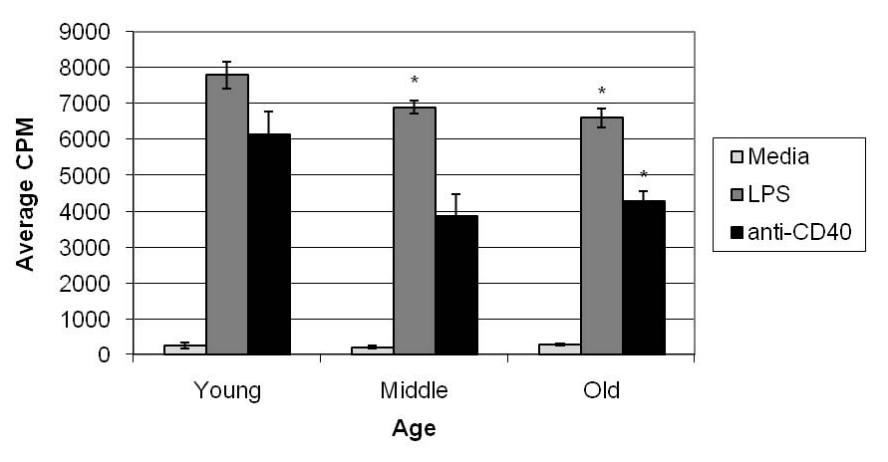
**Middle-aged and aged B cells show decreased ability to proliferate under stimulating conditions**. SPC from young, middle-aged, and aged QM mice were harvested and cultured in triplicate with anti-CD40 mAb or LPS for 24 hours and incubated with [3H]-Tdr and incubated another 24 hours. CPM was measured as described in material and methods. Error bars are shown for triplicate samples. Middle-aged and aged B cells stimulated with LPS show a significant decrease in proliferative capacity (P = 0.03 and 0.005 respectively). Aged B cells stimulated with LPS show a significant decrease (P = 0.029) while middle-aged cells appear to be decrease are not significantly different (P = 0.09). Data represents 4 experiments. P values determined by student's T-test.

Since blasts are formed prior to cell division, we considered that perhaps this trend of lower blast cell formation would have consequences in the ability of aged B cells to proliferate. Freshly isolated splenocytes from QM young and aged mice were cultured under stimulating conditions (LPS or anti-CD40 mAb) for 48 hours. Cells were allowed to incorporate [3H]-Tdr for 24 hours prior to harvest. Results indicate no significant difference between aged and young B cells when cultured in media (P > 0.05) (Figure 6). As predicted, however, aged B cells show a significant decrease in their proliferative capability in both LPS and anti-CD40 mAb stimulated cultures (P = 0.005 and 0.029 respectively). Middle-aged B cells also show a significant decrease in their ability to proliferate when stimulated with LPS (P = 0.031). A slight decrease was noted between middle-aged and young B cells when stimulated with anti-CD40 mAb but the difference was not significant. Thus, while aged B cells were able to respond to stimuli and proliferated above media levels, they were not as effective as young B cells.

## Discussion

Previous data from our lab has suggested that aged B cells were capable of undergoing normal activation events *in vivo *including germinal center reactions, but were defective in expansion following TD stimulation [29]. To investigate this further, lymphocytes were cultured *in vitro *with LPS to induce a TI response or anti-CD40 mAb to induce a TD response. Their ability to express activation markers, form blasts and proliferate was examined. Aged B cells were found to be dysfunctional in their ability to proliferate but were able to appropriately regulate expression of activation markers. Importantly, in all cases, stimulated aged B cells always showed a response above unstimulated cells suggesting some function is retained.

Song et al. [8] have reported that aged splenocytes appear to be comparable in expression and function of CD40 to their young counterparts while it has also been noted that the proportion of CD40^+ ^resting B cells remains relatively stable during ageing [8,27]. Contrary to these findings is the report by Lio et al. [25] in which aged peripheral blood mononuclear cells (PBMNC) showed reduced expression of CD40 and CD40L. It is unclear if this discrepancy is due to differences in microenvironment or age-related dysfunction. Our previous *in vivo *experiments utilized B cells from Ig transgenic (QM) mice containing a targeted gene insertion that allows the NP-specific B cells to retain the ability to participate in germinal center reactions [29,33,34]. The NP hapten is not an environmental antigen, thus aged QM mice contain a large population of antigen-inexperienced cells [33]. Due to environmental pressure, however, aged QM mice also develop a population of non-transgenic Tg cells derived from VH gene replacements which allowed us to observe both naïve and potentially antigen-experienced cells [32]. Because of the transgenic nature of the QM mice, all experiments were also conducted using normal C57 mice and no differences were noted between normal and transgenic mice. Our results showed equivalent expression of CD40 on all B cells examined. Thus, any age-associated dysregulation due to a defect within the CD40/CD154 interaction could not be attributed merely to a decrease in surface expression of the CD40 molecule.

To examine the functional consequences of engaging CD40, the ability of aged B cells to up or down-regulate early activation markers (CD86, CD69 and CD38) was tested. Our data suggest that cultured, aged murine B cells are responsive to stimulation, both LPS and CD40, with normal upregulation of CD86 and CD69 and down-regulation of CD38. It has been suggested that splenic B cells from aged GC of C57BL/6 mice lack CD86, however, this observation may be attributed to the poor T cell help provided in these experiments and not an intrinsic defect in the aged cells' ability to express this protein [35]. Another study, using aged B6D2F1 mice, showed that B cells from the GCs of Peyer's patches have normal expression of CD86 [36]. These reported differences in CD86 expression may be attributed to differences in experimental set-up including different strains of mice, immunization protocols and tissues used. Similar observations have been made with the expression of CD69. It has been noted that expression of CD69 on γδT lymphocytes is increased in elderly [37] while Herndandez-Garcia et al. found that expression of CD69 on peripheral blood lymphocytes in elderly was no different than in young [38].

Another early activation event induced by stimulation is the formation of blasts. We have found that aged B cells form blasts in response to LPS or anti-CD40 stimulation in all cases, however there was a consistent trend toward a lower percentage of induced blast cells. Unexpectedly, cells from individual aged donors cultured in medium alone frequently (about 50% of the time) demonstrated increased blast formation compared to young cells cultured under the same conditions. It is unclear why individual aged animals varied in this trait as there was no correlation to specific age (for example we observed this in both mice closer to 15 months and those closer to 19 months), gender (it occurred in both males and females), and the mice were genetically identical.

Proliferation of B cells was significantly decreased in aged animals. These data are consistent with studies showing that the GC reactions in aged animals are reduced in number and size [15,16]. It has been noted by Whisler et al [39] that aged human peripheral blood B cells consistently showed a 50% reduction in proliferation upon stimulation with *Staphylococcus aureus*, while Song et al [8] has shown that aged murine splenic cells cultured with anti-CD40 mAb proliferated more robustly than their young counterparts. The reasons for these differences are unclear. The current results from both the blast and proliferation assays indicate either the inability of aged B cells to respond to stimulation or an increase in apoptosis following stimulation. It is possible that aged and young cells undergo equal rounds of division but apoptosis may be induced more frequently in the aged cells. It will be of interest in future studies to examine the impact of decreased proliferative ability following primary antigen exposure on secondary antibody responses.

## Conclusion

The mechanisms of immune dysfunction in the elderly are unclear and an understanding of this dysfunction is becoming increasingly important as the average lifespan reaches record levels. We have shown that aged B cells are intrinsically dysfunctional and contribute to ineffective humoral immunity in the elderly. Other studies, particularly in mucosal immunity, support the notion of early activation defects, for example the proper homing to effector sites [30,31]. Surprisingly, the early response from aged B cells was not completely defective since, in all cases, at least a partial response was seen. This observation gives hope that effective therapies can be produced to help increase antibody responses. With high morbidity and mortality from infectious diseases, it is critical that age-related defects in immune responses be identified.

## Methods

### Mice

C57BL/6 mice were obtained from the National Cancer Institute (Bethesda, MD) and housed at Illinois State University's animal research facility under approval from the Institutional Animal Care and Use Committee (Protocol #3-2007). Quasi-monoclonal (QM) Ig-transgenic mice, kindly supplied by M. Cascalho, were developed using "knock-in" technology to produce a mouse with B cells specific for nitrophenyl hapten (NP) [33]. QM mice were backbred to the C57/BL6 J_H_^-/- ^J_k_^-/- ^strain to produce QMF9. Some QMF9 were bred for a single generation to C57BL/6 to produce QMKappa mice. A breeding colony of each strain is maintained at Illinois State University's animal research facility. Animals used for these experiments were either young (4–6 months), middle aged (11–13 months), or older (16–18 months).

### Cell Cultures

Spleens were removed aseptically from young, middle-aged, or aged QM mice and a single cell suspension was prepared in RPMI 1640 (GibCo Invitrogen Corporation, Brad Island, NY). Red blood cells were lysed with ACT buffer (0.144 M NH4Cl and 0.017 M Tris, pH 7.2). Lymphocytes were cultured at 3 × 10^6 ^cells/well in 6 well cell culture plates in CO2 incubator for 24–48 hours in complete RPMI media (RPMI supplemented with 10% fetal bovine serum (FBS; Hyclone; low endotoxin, low IgG), Penicillin G, L-glutamine, Streptomycin, 2-Mercaptoethanol, and 25 mM HEPES). Some cultures contained 50 μg/ml Lipopolysaccharide from Escherichia coli K-235 (LPS, Sigma Chemical, St. Louis, MO) or 10 μg/ml anti-CD40 mAb (1C10, Southern Biotechnology, Birmingham, AL) and 40 U/ml murine IL-4 (Peprotech, Rocky Hill, NJ).

### In vitro proliferation

Spleens were prepared as described above and 2 × 10^5 ^RBC-depleted splenocytes were cultured in 96 well microtiter plates in CO2 incubator for 24 hours in complete RPMI. Some cultures contained 50 μg/ml LPS or 10 μg/ml 1C10 and 20 U/ml murine IL-4. Tritiated thymidine ([3H]-Tdr) (1 μCi/well) was added and the cells were incubated an additional 24 hours. Cells were harvested through a PHD™ cell harvester (Cambridge Technology, Inc., Cambridge, MA) and samples run on a Beckman multi-purpose scintillation counter (Beckman Coulter, Inc., Fullerton, CA).

### Flow Cytometry

Freshly isolated or cultured cells were stained for flow cytometric analysis with saturating dilutions of anti-B220 Cy-Chrome, anti-CD38 biotin (Pharmingen, San Diego, CA), anti-CD69-PE, anti-CD86-FITC, streptavidin-PE (Southern Biotechnology, Birmingham, AL), anti-Id-FITC (kindly provided by T. Imanishi-Kari), or NP-PE (Biosearch Technologies, Novato, CA). Samples included 10% normal rat serum to prevent non-specific binding. Cells were analyzed immediately or fixed by adding 300 μl cold 0.5% paraformaldehyde in PBS and mixed thoroughly. Samples were analyzed on a Becton Dickinson FACSCalibur flow cytometer using CellQuest Pro software (San Jose, CA) with 10,000 events collected.

## Competing interests

The authors declare that they have no competing interests.

## Authors' contributions

KM set up the protocols and began the studies as well as performed the analysis of resting cell phenotypes, AB carried out the proliferation, phenotype and blast formation under stimulating conditions, while LV conceived the study, participated in its design, coordinated the experiments and helped draft the manuscript. All authors read and approved the final manuscript.
